# Supplementary material for: Green Extraction-Assisted Pseudo-Targeted Profile of Alkaloids in Lotus Seed Epicarp Based on UPLC-QTOF MS with IDA
Source: Foods. 2022 Apr 6;11(7):1056. doi: 10.3390/foods11071056 (PMC8997499; doi:10.3390/foods11071056)
Supplement: Supplementary file 1 [file foods-11-01056-s001.zip › foods-1645896-SI.pdf]

Supplementary Materials

Green extraction assisted pseudo-targeted profile of alkaloids in lotus seed epicarp based on UPLC-QTOF MS with IDA

Xiaoji Cao[1,2*], Xupin Lin[2], Congcong Wu[2], Minghua Zhang[2], Mingwei Wang[2]

[1] Research Center of Analysis and Measurement, Zhejiang University of Technology, Hangzhou 310014, Zhejiang, China; xiaojicao@zjut.edu.cn

[2] College of Chemical Engineering, Zhejiang University of Technology, Hangzhou 310014, Zhejiang, China; xupinl@163.com (X.L.); 807047075@qq.com (C.W.); 719838005@qq.com (M.Z.); 1002296149@qq.com (M.W.)

* Corresponding: xiaojicao@zjut.edu.cn (X.C.); Tel.: +86-0572-8813458 (X.C.)

S1 Single-factor experiments

When the ethyl lactate concentration (10, 30, 50, 70, 90, and 100% (v/v)) was selected as the control factor, other conditions were solid-to-liquid ratio of 1:20 g/mL, ultrasonic time of 10 min, ultrasonic temperature of 50 °C and ultrasonic power of 70 W. When the solid-to-liquid ration (1:5, 1:10, 1:15, 1:20, 1:25 and 1:30 g/mL) was used as the control factor, other conditions were ethyl lactate concentration of 50%, ultrasonic time of 10 min, ultrasonic temperature of 50 °C and ultrasonic power of 70 W. When the ultrasonic time (5, 10, 15, 20, 25 and 30 min) was used as the control factor, other conditions were ethyl lactate concentration of 50%, solid-to-liquid ratio of 1:20 g/mL, ultrasonic temperature of 50 °C and ultrasonic power of 70 W. When the ultrasonic temperature (30, 40, 50, 60 and 70 °C) was used as the control factor, other conditions were ethyl lactate concentration of 50%, solid-to-liquid ratio of 1:20 g/mL, ultrasonic time of 10 min and ultrasonic power of 70 W. When the ultrasonic power (50, 60, 70, 80, 90 and 100 W) was used as the control factor, other conditions were ethyl lactate concentration of 50%, solid-to-liquid ratio of 1:20 g/mL, ultrasonic time of 10 min, temperature of 50 °C.

S2 Establishment of the standard curve of nuciferine

A stock solution of nuciferine standard was prepared with methanol at a concentration of 0.1 mg/mL and stored at 4 °C. Working standard solutions were prepared covering a concentration range from 1 to 200 μg/L (1, 10, 50, 100, 150, 200 μg/L) for UPLC-MS/MS analysis. By external standard method, the standard curve was drawn with the vertical coordinate of the peak area intensity of nuciferine and the horizontal coordinate of the concentration. The standard curve equation of nuciferine was obtained as y=4193.67351x+336.02980 (R^2=0.999).

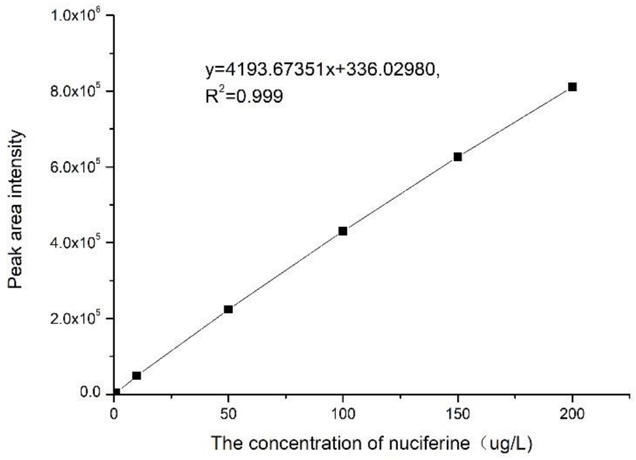

Figure S1. The standard curve of nuciferine

S3 Conventional reference extraction methods

(1) ultrasonic-assisted extraction with ethanol

1.0 g lotus seed epicarp powder and 25 mL ethanol aqueous solution (90%, v/v) were displaced in a 50 ml glass vial and extracted under ultrasound for 30 min, where the ultrasonic temperature was 50 ˚C and the ultrasonic power was 60 W.

(2) ultrasonic-assisted extraction with methanol

0.5 g lotus seed epicarp powder and 25 mL methanol aqueous solution (70%, v/v) were displaced in a 50 ml glass vial and extracted under ultrasound for 30 min, where the ultrasonic temperature was 50 ˚C and the ultrasonic power was 60 W.

(3) reflux with ethanol

1.0 g lotus seed epicarp powder and 10 mL ethanol aqueous solution (70%, v/v) were displaced in a 50 ml flask and boiled at 90 °C for 60 min under the oil-bath reflux (201D, Huichuang, Hangzhou, China).

(4) reflux with methanol

1.0 g lotus seed epicarp powder and 10 mL methanol aqueous solution (50%, v/v) were displaced in a 50 ml flask and extracted at 25 °C for 60 min under the oil-bath reflux (201D, Huichuang, Hangzhou, China).

Table S1. Factors and levels of variables for the Box-Behnken design.

Factors	Levels		
	-1	0	1
ethyl lactate concentration (%) - A	30	50	70
solid-to-liquid (g/mL) - B	1:10	1:15	1:20
ultrasonic time (min) - C	5	10	15

Table S2. The MRM^{HR} experiments parameters of 10 target alkaloids

Compounds name	Precursor ion (Da)	Fragment ion (Da)	Declustering potential(V)	Collision energy(V)
nicotinamide	123.05	80.0514	80	33
N-nornuciferine	282.14	265.1235	80	20
coclaurine	286.14	107.0491	80	31
N-methycoclaurine	300.16	107.0491	80	33
nuciferine	296.16	265.1210	80	27
asimilobine	268.13	251.1074	80	22
isoliensinine	611.31	192.1008	80	40
neferine	625.33	206.1158	80	40
cis -*N*-feruloyltyramine	314.13	145.0290	80	35
cis/tran-*N*-feruloyltyramine	314.13	145.0290	80	35

Table S3. The result of 17 experimental groups for RSM

Run	A	B	C	Total alkaloids content (mg nuciferine/g)
1	-1	-1	0	26.02
2	1	-1	0	32.20
3	-1	1	0	28.45
4	1	1	0	31.47
5	-1	0	-1	27.72
6	1	0	-1	31.42
7	-1	0	1	26.31
8	1	0	1	33.45
9	0	-1	-1	33.63
10	0	1	-1	34.72
11	0	-1	1	33.55
12	0	1	1	34.09
13	0	0	0	33.29
14	0	0	0	33.22
15	0	0	0	32.36
16	0	0	0	32.65
17	0	0	0	32.37

Table S4. Analysis of variance (ANOVA) for quadratic model

Source	Sum of Squares	df	Mean square	F-Value	p-Value	
Model	118.20	9	13.13	67.27	< 0.0001	Significant
A	50.23	1	50.23	257.27	< 0.0001	
B	1.40	1	1.40	7.15	0.0318	
C	0.001246	1	0.001246	0.006381	0.9386	
AB	2.51	1	2.51	12.84	0.0089	
AC	2.96	1	2.96	15.16	0.0059	
BC	0.078	1	0.078	0.40	0.5465	
A^2	59.45	1	59.45	304.49	< 0.0001	
B^2	1.11	1	1.11	5.69	0.0485	
C^2	2.08	1	2.08	10.67	0.0138	
Error	1.37	7	0.20			
Lack of Fit	0.55	3	0.18	0.91	0.5130	not significant
Pure Error	0.81	4				
Cor Total	119.57	16				
R^2	0.9866					
Adj R^2	0.9739					

Table S5. Characterization of alkaloids in lotus seed epicarp by UPLC-QTOF-MS/MS in positive mode

NO.	t_R (min)	$[M+H]^+$ measured	M	Molecular formula	MS^2 fragment ion （*m/z*）	Identification
1	0.78	123.0552	122.0475	$C_6H_6N_2O$	106.0286, 96.0443, 80.0514, 78.0360	Nicotinamide
2	0.90	434.1809	433.1736	$C_{22}H_{27}NO_8$	272.1268, 255.1009, 161.0578, 143.0473, 107.0475	norcoclaurine-4’-*O*-glucoside
3	0.95	448.1965	447.1887	$C_{23}H_{29}NO_8$	286.1439, 255.1018, 161.0596, 107.0491	*N*-methylnorcoclaurine 7-*O*-glucoside
4	1.12	448.1965	447.1887	$C_{23}H_{29}NO_8$	286.1439, 255.1018, 161.0596, 107.0491	*N*-methylnorcoclaurine 4’-*O*-glucoside
5	1.25	462.2122	461.2044	$C_{24}H_{31}NO_8$	300.1600, 269.1178, 257.1180, 107.0487	*N*-methylcoclaurine 7-*O*-glucoside
6	1.33	448.1965	447.1887	$C_{23}H_{29}NO_8$	286.1438, 269.1173, 237.0905, 209.0952, 107.0497	coclaurine 7-*O*-glucoside
7	1.55	448.1965	447.1887	$C_{23}H_{29}NO_8$	286.1438, 269.1173, 237.0905, 209.0952, 107.0497	isococlaurine 4’-*O*-glucoside
8	1.56	462.2122	461.2044	$C_{24}H_{31}NO_8$	300.1600, 269.1178, 257.1180, 107.0487	*N*-methylcoclaurine 4’-*O*-glucoside
9	1.67	298.1438	297.1360	$C_{18}H_{19}NO_3$	255.1033, 237.0899, 223.0740, 195.0788	glaziovine
10	1.77	272.1282	271.1208	$C_{16}H_{17}NO_3$	255.1016, 161.0507, 143.0411, 123.0377, 107.0491	norcoclaurine
11	1.86	286.1438	285.1360	$C_{17}H_{19}NO_3$	255.1019, 209.0948, 161.0593, 143.0487, 107.0486	*N*-methylnorcoclaurine
12	2.01	476.2278		$C_{25}H_{33}NO_8$	314.1755, 283.1336, 107.0496	lotusine 7-*O*-glucoside
13	2.56	314.1751		$C_{19}H_{23}NO_3$	269.1172, 237.0910, 107.0484	lotusine
14	2.71	476.2278		$C_{25}H_{33}NO_8$	314.1755, 283.1336, 107.0496	lotusine 4’-*O*-glucoside

15	3.66	300.1594	299.1521	$C_{18}H_{21}NO_3$	269.1165, 237.0911, 209.0951, 175.0745, 143.0488, 107.0496	*N*-methylisococlaurine
16	4.05	286.1438	285.1360	$C_{17}H_{19}NO_3$	269.1173, 237.0905, 209.0952, 175.0750, 143.0487, 107.0497	coclaurine
17	4.29	300.1594	299.1521	$C_{18}H_{21}NO_3$	269.1165, 237.0911, 209.0951, 175.0745, 143.0488, 107.0496	*N*-methylcoclaurine
18	4.53	312.1594	311.1516	$C_{19}H_{21}NO_3$	283.1326, 269.1164, 254.0937, 238.0991, 223.0740, 195.0788	pronuciferine
19	5.12	314.1751	313.1673	$C_{19}H_{23}NO_3$	283.1330, 268.1096, 252.1147, 237.0909, 151.0754, 107.0491	armepavine
20	5.16	300.1594	299.1516	$C_{18}H_{21}NO_3$	283.1343, 268.1097, 237.0907, 189.0807, 107.0495	*N*-norarmepavine
21	5.17	611.3120	610.3043	$C_{37}H_{42}N2O_6$	580.2686, 568.2684, 475.2215, 192.1008, 121.0636	isoliensinine
22	5.30	286.1438	285.1360	$C_{17}H_{19}NO_3$	269.1173, 237.0905, 209.0952, 175.0750, 143.0487, 107.0497	isococlaurine
23	5.31	300.1594	299.1521	$C_{18}H_{21}NO_3$	269.1165, 237.0911, 209.0951,175.0745, 143.0488, 107.0496	6-demethyl-4'-methyl-*N*-methylcoclaurine
24	5.37	268.1332	267.1254	$C_{17}H_{17}NO_2$	251.1074, 236.0840, 219.0809, 191.0856	asimilobine
25	5.38	625.3272	624.3199	$C_{38}H_{44}N_2O_6$	594.2864, 503.2546, 489.2392, 206.1181	neferine
26	5.48	282.1489	281.1411	$C_{18}H_{19}NO_2$	251.1066, 236.0838, 219.0800, 191.0853	*O*-nornuciferine
27	5.49	312.1594	311.1516	$C_{19}H_{21}NO_3$	281.1158, 266.0926, 250.0976, 235.0692	oxidation-nuciferine
28	5.51	314.1751	313.1673	$C_{19}H_{23}NO_3$	283.1322, 251.1065, 175.0772, 121.0651	4'-methyl-*N*-methylcoclaurine
29	5.61	268.1332	267.1254	$C_{17}H_{17}NO_2$	251.1074, 236.0840, 219.0809, 201.0694, 191.0856	caaverine
30	5.64	298.1438	297.1360	$C_{18}H_{19}NO_3$	251.1074, 236.0840, 219.0809, 191.0856	*N*-methyl asimilobine-*N*-oxide
31	5.69	282.1489	281.1411	$C_{18}H_{19}NO_2$	251.1066, 236.0838, 219.0800, 201.0698, 191.0853, 165.0694	lirinidine

32	5.98	266.1175	265.1103	$C_{17}H_{15}NO_2$	249.0910, 219.0804, 191.0855	anonaine
33	6.00	282.1489	281.1411	$C_{18}H_{19}NO_2$	265.1219, 250.0980, 235.0747, 207.0814, 191.0853 ,165.0694	*N*-nornuciferine
34	6.03	280.1332	279.1254	$C_{18}H_{17}NO_2$	249.0911, 219.0801, 201.0605, 191.0854, 178.0770, 165.0697	roemerine
35	6.05	296.1645	295.1567	$C_{19}H_{21}NO_2$	265.1210, 250.0977, 235.0750, 219.0799, 191.0741, 165.0696	nuciferine
36	6.06	314.1387	313.1314	$C_{18}H_{19}NO_4$	177.0549, 145.0285, 121.0651, 103.0543	cis-*N*-feruloyltyramine
37	6.19	312.1594	311.1516	$C_{19}H_{21}NO_3$	265.1216, 250.0987, 235.0751, 219.0663, 191.0741	nuciferine-*N*-methanol
38	6.18	314.1387	313.1314	$C_{18}H_{19}NO_4$	177.0549, 145.0285, 121.0651, 103.0543	trans-*N*-feruloyltyramine
39	6.70	268.1332	267.1254	$C_{17}H_{17}NO_2$	131.0494, 121.0653, 103.0548	cis/trans isomers of *N*-cinnamoyltyramine
40	6.91	268.1332	267.1254	$C_{17}H_{17}NO_2$	131.0494, 121.0653, 103.0548	cis/trans isomers of *N*-cinnamoyltyramine
41	7.87	324.1594	323.1516	$C_{20}H_{21}NO_3$	265.1228, 250.1053, 233.0961, 218.0728	nuciferine-*N*-acetyl
42	7.97	308.1281	307.1203	$C_{19}H_{17}NO_3$	249.0906, 219.0802, 191.0850,178.0772, 165.0690	anonaine -*N*-acetyl

Table S6. Method validation of the ten alkaloids in lotus seed epicarp

Alkaloids	Liner range (μg/L)	Calibration curve	R^2	LOD (μg/L)	LOQ (μg/L)	RSD（%）（n=5）	
						Intra-day	Inter-day
nicotinamide	5-200	y=194.52378x-10.79699	0.999	0.52	2.57	1.95	2.66
N-nornuciferine	1-200	y=2108.24132x+255.27772	0.999	0.10	0.30	0.68	4.15
coclaurine	1-200	y=1075.04641x+374.17352	0.998	0.12	0.60	1.64	2.00
N-methycoclaurine	1-200	y=971.13868x+329.84089	0.996	0.14	0.70	0.68	1.61
nuciferine	1-200	y=4193.67351x+336.02980	0.998	0.10	0.35	2.91	4.40
asimilobine	1-200	y=908.27870x+29.76412	0.999	0.26	0.65	1.93	2.07
isoliensinine	5-200	y=213.92071x+0.50564	0.999	1.00	3.00	4.79	5.26
neferine	1-200	y=1152.56857+282.30732	0.998	0.36	1.00	2.62	4.02
cis-*N*-feruloyltyramine	1-200	y=327.46671x+96.65130	0.999	0.12	0.60	2.38	3.06
trans-*N*-feruloyltyramine	1-200	y=109.13443x+1.25007	0.999	0.25	0.50	0.97	2.74

Table S7. Recovery yields of the ten alkaloids in lotus seed epicarp

Alkaloids	Add (ng/mL)	Recovery (%)	RSD (%)
nicotinamide	5	93.71	5.33
	50	101.46	4.62
	100	101.43	4.34
N-nornuciferine	5	95.57	0.82
	50	90.13	0.80
	100	92.20	1.82
coclaurine	5	101.03	3.19
	50	102.03	2.65
	100	97.46	1.66
N-methycoclaurine	5	99.74	4.47
	50	99.52	3.40
	100	99.63	2.09
nuciferine	5	86.33	4.67
	50	89.83	3.85
	100	90.92	2.54
asimilobine	5	89.26	1.17
	50	90.97	1.56
	199	89.17	1.18
isoliensinine	5	93.20	7.38
	50	91.96	8.08
	100	96.44	5.94
neferine	5	93.63	3.43
	50	99.98	7.80
	100	87.87	4.88
cis-*N*-feruloyltyramine	5	81.09	4.78
	50	82.84	2.22
	100	89.13	2.47
trans-*N*-feruloyltyramine	5	92.58	7.64
	50	85.25	5.75
	100	86.22	8.75

Table S8. Contents of ten alkaloids in lotus seed epicarp at different growth stages

Stages\(μg/g)	I	II	III	IV	V
nicotinamide	1121.60±5.80	472.87±2.97	302.27±1.70	443.80±3.80	341.07±8.28
N-nornuciferine	314.20±6.79	1949.00±13.72	4555.33± 26.40	3356.67±32.53	1091.07±9.90
coclaurine	59.33± 8.07	369.67±9.90	978.27±8.34	673.67±11.03	120.64± 6.72
N-methycoclaurine	233.53±4.61	2034.13±2.83	1718.53±5.75	1348.00±4.38	1704.20±7.50
nuciferine	352.20± 12.16	2097.33±31.11	2118.67±14.14	2534.67±20.27	969.33± 4.90
asimilobine	84.00± 6.53	147.59±1.64	373.80±2.55	1108.80±11.31	588.93± 8.91
isoliensinine	26.86±3.14	22.53±1.99	109.59±7.25	186.39±4.78	112.26± 4.38
neferine	4.54± 0.14	2.35±0.42	16.75±1.46	15.49±2.55	9.77±0.07
cis-*N*-feruloyltyramine	-	42.82±1.10	7.06±0.01	-	-
trans-*N*-feruloyltyramine	40.55±1.62	52.30±3.25	97.27±6.13	478.40±7.35	19.25±2.80
Total alkaloids	2236.81±48.86	7190.59±68.93	10277.54±73.73	10145.89±98.0	4956.52±53.46
